# Increased DNA Copy Number Variation Mosaicism in Elderly Human Brain

**DOI:** 10.1155/2018/2406170

**Published:** 2018-06-28

**Authors:** Darine Villela, Claudia K. Suemoto, Renata Leite, Carlos Augusto Pasqualucci, Lea T. Grinberg, Peter Pearson, Carla Rosenberg

**Affiliations:** ^1^Human Genome and Stem Cells Research Center, Department of Genetics and Evolutionary Biology, Institute of Biosciences, University of São Paulo, Rua do Matão 277, 05508-090 São Paulo, SP, Brazil; ^2^Discipline of Geriatrics, Department of Internal Medicine, University of São Paulo Medical School, Avenida Doutor Arnaldo 455, 01246-000 São Paulo, SP, Brazil; ^3^Brazilian Aging Brain Study Group-LIM22, Department of Pathology, University of São Paulo Medical School, Avenida Doutor Arnaldo 455, 01246-000 São Paulo, SP, Brazil; ^4^Department of Pathology, University of São Paulo Medical School, Avenida Doutor Arnaldo 455, 01246-000 São Paulo, SP, Brazil; ^5^Memory and Aging Center, Department of Neurology, University of California, San Francisco 675 Nelson Rising Lane, P.O. Box 1207, San Francisco, CA 94143, USA

## Abstract

Aging is a complex process strongly determined by genetics. Previous reports have shown that the genome of neuronal cells displays somatic genomic mosaicism including DNA copy number variations (CNVs). CNVs represent a significant source of genetic variation in the human genome and have been implicated in several disorders and complex traits, representing a potential mechanism that contributes to neuronal diversity and the etiology of several neurological diseases and provides new insights into the normal, complex functions of the brain. Nonetheless, the features of somatic CNV mosaicism in nondiseased elderly brains have not been investigated. In the present study, we demonstrate a highly significant increase in the number of CNVs in nondiseased elderly brains compared to the blood. In two neural tissues isolated from paired *postmortem* samples (same individuals), we found a significant increase in the frequency of deletions in both brain areas, namely, the frontal cortex and cerebellum. Also, deletions were found to be significantly larger when present only in the cerebellum. The sizes of the variants described here were in the 150–760 kb range, and importantly, nearly all of them were present in the Database of Genomic Variants (common variants). Nearly all evidence of genome structural variation in human brains comes from studies detecting changes in single cells which were interpreted as derived from independent, isolated mutational events. The observations based on array-CGH analysis indicate the existence of an extensive clonal mosaicism of CNVs within and between the human brains revealing a different type of variation that had not been previously characterized.

## 1. Introduction

Aging is a complex process that involves altered cellular function, oxidative stress, longevity, and related diseases [1]. An important challenge for future research is to understand how genetics influences cognition and the neurobiological mechanisms underlying normal and pathological aging. It is expected that genetic variants may contribute to the considerable individual differences in cognitive aging by altering brain plasticity [2, 3]. Interestingly, previous reports have shown that individual neurons display somatic genomic mosaicism [4]. Although the effects of this somatic mosaicism are not fully understood, it is expected that alterations in the genome of neuronal cells will influence both the normal and the diseased brains. The forms of somatic genomic mosaicism identified in the human brain include aneuploidy [5], mobile genetic element insertions (MEIs) [6, 7], single-nucleotide variants (SNVs), indels [8], and, more recently, DNA copy number variations (CNVs) [4, 9].

CNVs represent a prevalent form of genetic variation that contributes to phenotypic diversity, numerous diseases, and complex traits in human populations [10]. The mechanisms by which copy number changes may affect gene expression, and phenotypic traits comprise alteration of gene dosage and disruption of coding sequences or regulatory elements [10]. McConnell and colleagues were the first to demonstrate increased levels of CNVs in cortical neurons derived from *postmortem* specimens and human-induced pluripotent stem cell (hiPSC) fibroblast-derived neurons compared to blood samples using a single-cell sequencing strategy [11]. The authors identified in both samples a subset of aneuploid neurons as well as numerous subchromosomal CNVs. Because of the long lifespan of neurons and their central role in synapses, it is speculated that the accumulation of somatic mutations within neural progenitor cells or in postmitotic neurons could influence neuronal development, complexity, and function. In this regard, genomic mosaicism in single, sporadic Alzheimer's disease neurons characterized by an increase in total DNA content and amyloid precursor protein (*APP*) gene copy number was reported [12]. The authors also showed large differences in the total DNA content between different brain areas, which may indicate distinct functionality for genomic mosaicism in the central nervous system.

The existence of region-specific somatic mosaicism of DNA content was also demonstrated in nondiseased human brains, suggesting that sporadic brain diseases may depend on which pathogenic loci are altered [12, 13]. However, the features of mosaic CNV in nondiseased elderly brains have not been characterized. It is not clear yet when and what promotes CNV formation in human brains or how does it relate to aging. Moreover, it is worth to mention that nearly all evidence of genome structural variation in human brains comes from studies detecting changes in single cells, either by FISH for detection of chromosomal aneuploidy or by total DNA sequencing of single cells for chromosomal structural rearrangements. The genomic changes observed in single cells were interpreted as derived from independent, isolated mutational events. In this study, based on a comparative array-comparative genomic hybridization (array-CGH) analysis of two brain tissues and blood-isolated *postmortem* samples from the same individuals, we report the existence of an extensive clonal mosaicism for CNV in and between the cerebellum and the frontal cortex compared to the blood, which probably reflects a higher mutation rate in neural tissues.

## 2. Materials and Methods

### 2.1. Postmortem DNA Samples

All the samples used in this study were provided by the Brain Bank of the Brazilian Aging Brain Study Group (BBBABSG) [14, 15]. The clinical and functional status of all subjects was assessed through the closest family member to the deceased who completed questionnaires on whether or not the subject was demented or suffered from other possible neural conditions, including a previous history of stroke, epilepsy, or Parkinson's disease. All questionnaires were based on a validated clinical protocol that includes a series of semistructured scales covering major functional abilities [16, 17] and cognitive evaluation by the Clinical Dementia Rating Scale [18] and the Informant Questionnaire on Cognitive Decline in the Elderly [19]. As a standard protocol for neuropathological diagnosis, the brain was examined macroscopically, and 15 brain regions were sampled for microscopic evaluation. Neuropathological examinations were carried out using immunohistochemistry following internationally accepted guidelines [20–23]. BBBABSG's procedures are approved by the Ethical Board of the University of São Paulo Medical School, and the next of kin agreed to participate and signed an informed written consent. Initially, investigations on CNV frequencies were derived from array-CGH data taken from 24 blood and 71 cerebellum independent samples (different individuals). Subsequently, 19 paired blood/cerebellum samples from the same individuals were used to confirm the differences in CNV frequencies between the two tissues. In addition to the paired samples from 19 individuals, we obtained matched frontal cortex tissues from 10 of the individuals.  Table 1 presents the characterization of all individuals classified from the paired analysis cohort.

### 2.2. Array-CGH

CNVs were identified using comparative genomic hybridization based on microarrays (array-CGH) containing 180,000 oligonucleotides (Oxford Gene Technologies, UK). Briefly, samples were labeled with Cy3- and Cy5-deoxycytidine triphosphates by random priming. Purification, hybridization, and washing were carried out as previously reported [24]. Scanned images of the arrays were processed using Feature Extraction software, and data were analyzed with the Genomic Workbench software, both software from Agilent Technologies (Santa Clara, CA, USA). CNVs were identified using the aberration detection method 2 statistical algorithm (ADM2) with a sensitivity threshold of 6.7. A genomic segment was considered duplicated or deleted when the log_2_ ratio of the test/reference fluorescent intensities of a given region encompassing at least three probes which were above 0.3 or below −0.3, respectively. The equivalency between the log_2_ ratios of the test/reference for duplications is 0.58 and for deletions is −1. Detected CNVs were compared to CNV data from oligoarray studies documented in the Database of Genomic Variants (DGV).

### 2.3. Statistical Analyses

Data are presented as the mean ± SEM. Statistical analyses (GraphPad Prism 6.0 software, San Diego, CA, USA) were performed using the nonparametric Mann–Whitney *U* test for the comparison between two groups. One-way ANOVA followed by Bonferroni's post hoc test was applied to estimate the mean differences between three groups. Two-way ANOVA was used to compare the mean differences between groups with two independent variables.

## 3. Results

**Figure 1:**
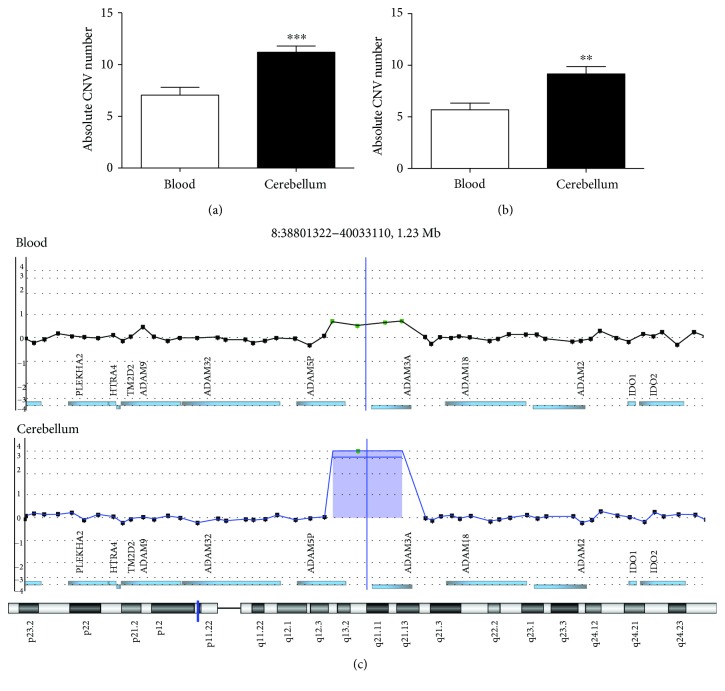
Increase frequency of DNA copy number variation (CNVs) in neural tissue compared to the blood. (a) The mean of the total CNVs detected in independent blood (*n* = 24) and cerebellum tissue (*n* = 71) samples; ^∗∗∗^ *p* < 0.0001, Mann–Whitney *U* test. (b) The mean of the total CNVs detected in paired blood and cerebellum samples from 19 random individuals; ^∗∗^ *p* < 0.001, Mann–Whitney *U* test. (c) Example of a CNV absent in the blood and observed in the cerebellum. Images extracted from Genomic Workbench software.

The investigation of CNVs by array-CGH in independent samples of nondemented elderly individuals revealed a highly significant increase in the frequency of CNVs in *postmortem* samples from the cerebellum (*n* = 71) compared to blood samples (*n* = 24) (Mann–Whitney *U* test, *p* < 0.0001) (Figure 1(a)). This finding was later confirmed in 19 paired blood/cerebellum samples taken from the same individuals (Mann–Whitney *U* test, *p* < 0.001) (Figure 1(b)).  Figure 1(c) shows an example of many more copies of a segment of chromosome 8 in the cerebellum than in the blood. For some of the paired cerebellum/blood samples, we were also able to obtain matched frontal cortex tissue and determine the distribution of CNVs between these two neural tissues within the same individuals.

**Figure 2:**
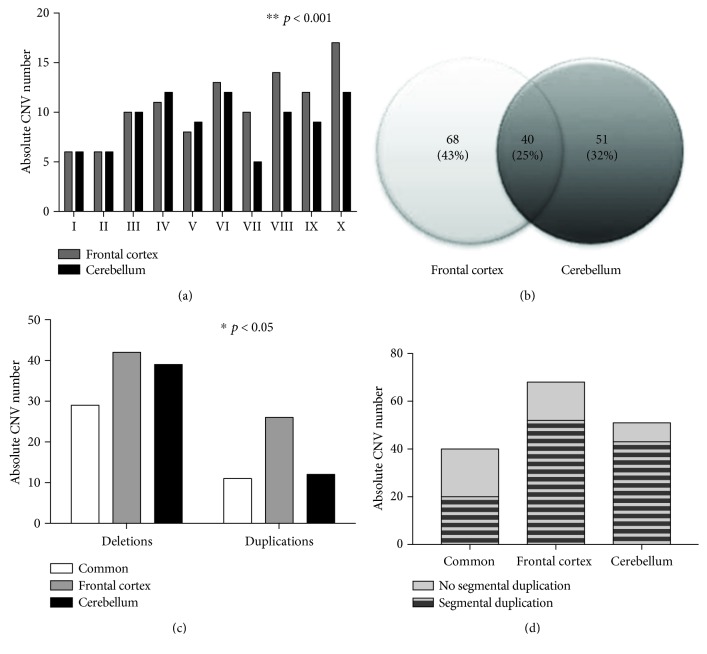
Distribution of DNA copy number variations (CNVs) between two neural tissues from the same individuals. (a) Comparison of the absolute number of CNVs detected in the frontal cortex and cerebellum from 10 paired individuals; ^∗∗^ *p* < 0.001 for comparison between individuals, two-way ANOVA. (b) Venn diagram showing the proportion of CNVs observed in the frontal cortex and cerebellum. (c) Total number of deletions and duplications observed in the CNVs in common to both neural tissues and in the unique CNVs from the frontal cortex and cerebellum; ^∗^ *p* < 0.05 for comparisons between the compared groups as well as deletions and duplications, two-way ANOVA. (d) Proportion of segmental duplications identified in the CNVs in common to both neural tissues and in the unique CNVs from the frontal cortex and cerebellum.

The distribution of absolute CNV numbers in the frontal cortex and cerebellum revealed high heterogeneity both between individuals and tissues (two-way ANOVA, *p* < 0.001) (Figure 2(a)). Further inspection using a Venn diagram (Figure 2(b)) shows the proportion of CNVs observed exclusively either in the frontal cortex or in the cerebellum (referred to as unique CNVs). In total, 75% of the CNVs correspond to unique mutations to one of these two neural tissues (43% in the frontal cortex and 32% in the cerebellum). The remaining 25% refers to CNVs in common to both tissues, suggesting that they were acquired earlier during brain development. All the 159 CNVs identified in these two brain areas are presented in Supplementary Table 1. We also evaluated the CNVs regarding the frequency of deletions and duplications, their length, and gene content. For both in common and in unique CNVs from the frontal cortex and cerebellum, the frequency of deletions was higher than that of duplications (two-way ANOVA, *p* < 0.05, Figure 2(c)). Comparing the lengths of the CNVs, the mean sizes of duplications and deletions differed significantly among groups (^∗^ *p* < 0.05, one-way ANOVA), and the unique deletions from the cerebellum were significantly larger than the ones in common (Bonferroni's test, *p* < 0.05). Nonetheless, in the present data, neither the length nor gene content of the unique CNVs from the frontal cortex and cerebellum areas were significantly different from each other (Table 2).

**Figure 3:**
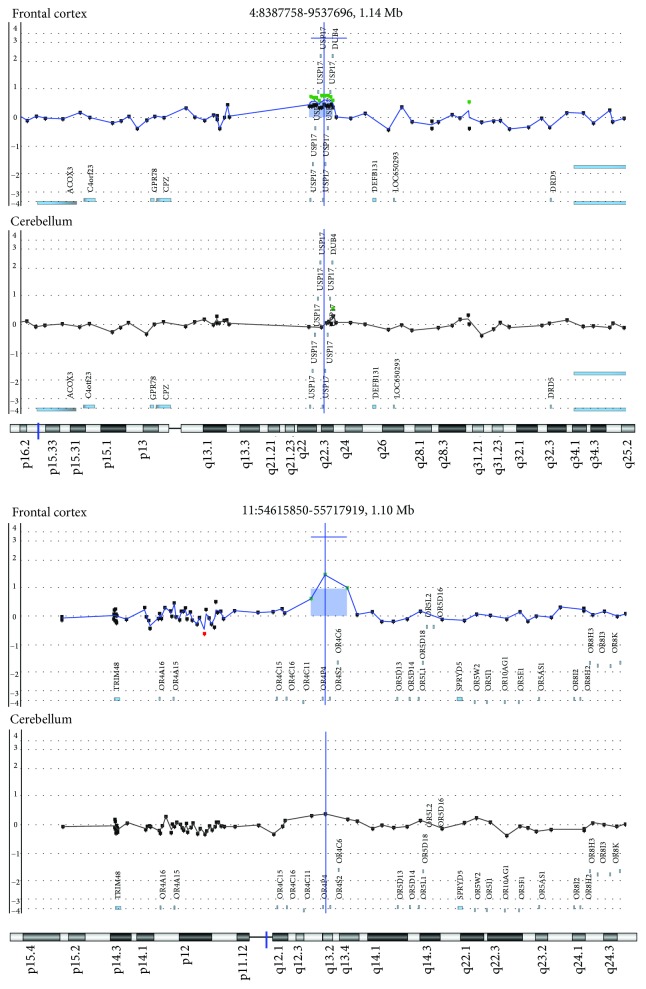
Unique DNA copy number variations (CNVs) from neural tissues. Two examples of somatic duplications that are present in the frontal cortex but absent in the cerebellum. Images extracted from Genomic Workbench software.

Importantly, nearly all the observed CNVs in this study, either present in both brain tissues or exclusively to one of them, had already been described in the Database of Genomic Variants (DGV) as polymorphic, being most of them segmental duplications (Figure 2(d)).  Figure 3 shows two examples of somatic duplications that are present in the frontal cortex but absent in the cerebellum. As in most of the CNVs observed in this study, the amplified segment involves a gene family with variable copy number variation in the population.

**Figure 4:**
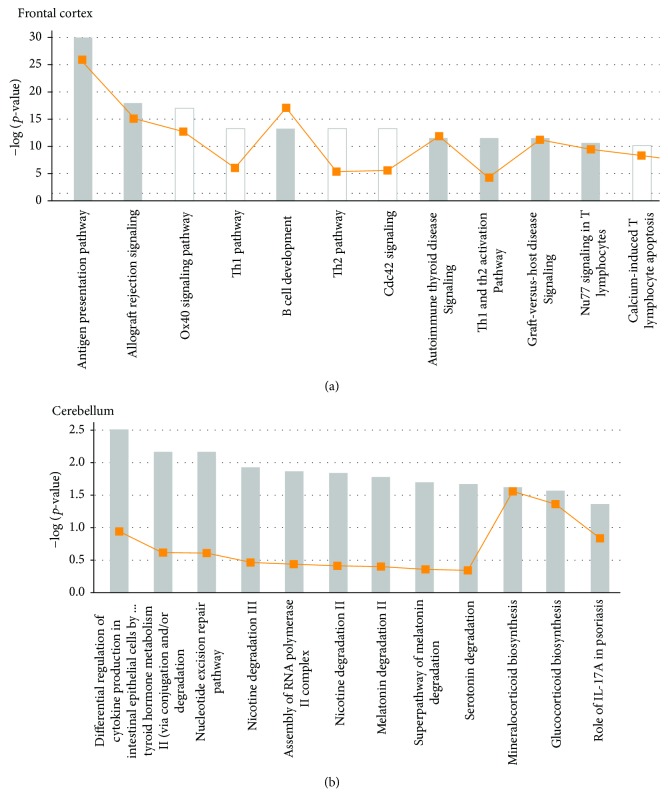
Top canonical pathways identified in ingenuity pathway analysis (IPA). The *x*-axis represents the top canonical pathways calculated by IPA based on all DNA copy number variations (CNVs) identified in the frontal cortex (a) and cerebellum (b). The yellow line represents the threshold of *p* value < 0.05 as calculated by Fischer's test.

Using the ingenuity pathway analysis (IPA) software (QIAGEN Inc., https://www.qiagenbioinformatics.com/products/ingenuitypathway-analysis), we retrieved the predicted target pathways of all the CNVs identified in the frontal cortex and cerebellum and found enrichment for antigen presentation and regulation cytokine production pathways, respectively (Figure 4); the top diseases/function of all networks retrieved from both neural tissues are presented in Supplementary Table 2.

## 4. Discussion

Several studies have demonstrated that neuronal genomes exhibit somatic genomic mosaicism compared to other tissues including CNVs [4]. Notably, genome structural variation in the human brain has been reported in single cell studies, using either FISH or DNA sequencing involving large-scale changes such as aneuploidy and structural rearrangements of more than 7 Mb [11, 25]. Our study is the first to investigate the clonal CNV burden within different neural tissues in nondiseased elderly brains using direct microarray analysis.

In contrast to previous studies on single cells using either FISH or DNA sequencing, our results, based on array-CGH analysis, represent genomic changes in DNA pools from a large number of cells from each tissue investigated, which indicates that at least some of these increase in CNV variation in human brains relative to the blood must occur as extensive clones rather than isolated events. The presence of extensive clones involving different CNVs indicates that these CNVs must either have been present in the germline (constitutive) or have originated early in embryonic development and could be distinguished from partial chromosome aneuploidies or large copy number variations as detected by FISH and/or sequencing of single cells [11, 26, 27]. The frequency of unique CNVs in each tissue is probably proportional to the CNV mutation rate for each tissue and should reflect CNV mutation rate differences between tissues. Significantly, nearly all the observed CNVs, both CNVs in common to both neural tissues and presumptively new mutations, had already been described in the Database of Genomic Variants (DGV) as polymorphic, and the amplified segment involves a gene family with variable copy number variation in the population, being most of them segmental duplications. This evidence suggests that many of these variants arose commonly and perhaps involve similar mechanisms in their origin, the nonallelic homologous recombination [10].

The CNV distribution within different human brain areas was highly heterogeneous both between individuals and tissues. A previous study based on flow cytometry demonstrated that the frontal cortex exhibits more variation in DNA content than the cerebellum [13] Although we did not quantify variation in DNA content across our DNA samples from the frontal cortex and cerebellum, we examined whether such differences in DNA content described by Westra et al. might be caused by increased rates of deletion and/or duplication in the frontal cortex compared to the cerebellum. Albeit not statistically significant, we observed an increase in the frequency only of duplications in the frontal cortex compared to the cerebellum. However, neither the length nor gene content of the unique CNVs from either these two neural tissues were significantly different from each other. Although Westra et al. reported that the variation in DNA content averaged ~250 Mb more DNA in the frontal cortex compared to the cerebellum by flow cytometry, this difference could not be directly related to DNA copy number variation. Further, their results are possibly explicable by a much higher involvement of retrotransposition in the neural cortex than the cerebellum culminating in a significantly wider variation in DNA content, similar to the differences induced by a higher number of L1 transpositions in the human hippocampus compared to the caudate nucleus [6].

IPA analysis was performed to investigate whether this increase of CNVs in the brain could be explained by an enrichment of pathways specific for central nervous system function. Based on the fact that the predicted target pathways of all brain CNVs are not specific for the central nervous system functioning, our array-CGH analyses indicate either an increased genomic instability or a less stringent selection against genomic imbalances in brain tissues compared to the blood, resulting in an increased frequency of clonal CNVs in neural tissues. Nonetheless, the exact mechanisms leading to such neuronal genomic instability are still a matter of speculation, but retrotransposon insertion has been demonstrated to mediate the formation of CNVs in various brain tissues [6] and also to involve double-strand DNA breaks [28]. Even so, CNVs represent a significant source of genetic variation in the human genome and have been implicated in several disorders and complex traits, representing a potential mechanism that contributes to neuronal diversity. Besides, the highly significant increase of CNVs that we reported here potentially contributes to physiologic variability and neuronal plasticity and can provide new insights into the complex functioning of the brain.

In summary, this study demonstrates an extensive clonal mosaicism for copy number variation between two different brain tissues. The investigation of genomic changes based on tissue DNA instead of single cells revealed a different type of variation than previously reported. The variation detected in tissue (clonal) seems to involve smaller segments, which are variable in number in the population, likely having a smaller phenotypic impact than the aneuploidies or chromosome alterations seen in single cells. Even so, they reflect a higher genomic instability or less stringent selection in the brain than in the blood, which deserves attention.

## Figures and Tables

**Table 1:** Clinical data of all individuals classified as nondemented included in the paired analysis cohort.

Case	Sex	Age at death	Schooling (years of formal education)	Neuropathological diagnosis	Cause of death
1	M	83	14	Normal	Ischemic cardiomyopathy
2	F	97	4	Normal	Dilated cardiomyopathy
3	F	73	12	Normal	Acute infarction myocardial
4	F	77	3	Normal	Tromboembolism pulmonar
5	M	64	4	Normal	Acute infarction myocardial
6	F	66	4	Normal	Healed myocardial infarction
7	F	67	4	Normal	Bilateral bronchopneumonia
8	F	62	1	Normal	Hemopericardium
9	F	70	4	Normal	Myocarditis
10	M	63	11	Normal	Acute infarction myocardial
11	F	81	11	Normal	Pulmonary edema
12	F	77	2	Normal	Hemopericardium
13	M	81	13	Normal	Bronchopneumonia
14	F	65	11	Normal	Acute infarction myocardial
15	M	76	4	Normal	Acute lung edema/myocardiopathy
16	F	75	4	Normal	Acute lung edema
17	M	50	13	Normal	Hypertensive cardiopathy
18	F	89	8	Normal	Acute infarction myocardial
19	M	85	2	Normal	Pulmonary edema

**Table 2:** Comparison of length and gene content of the in common and unique CNVs from the frontal cortex and cerebellum.

Copy number variation	CNVs in common to both tissues	Unique CNVs (frontal cortex)	Unique CNVs (cerebellum)
Mean size of CNVs (kb)	319 ± 85/*n* = 40	295 ± 60/*n* = 68	436 ± 84/*n* = 51
Deletions (Kb) (*p* < 0.05)^∗^^#^	151 ± 32/*n* = 29	299 ± 88/*n* = 42	478 ± 103/*n* = 39
Duplications (kb) (*p* < 0.05)^∗^	760 ± 261/*n* = 11	290 ± 72/*n* = 26	301 ± 125/*n* = 12
Gene content (kb)	1.33 ± 0.23/*n* = 40	1.57 ± 0.19/*n* = 68	1.38 ± 0.27/*n* = 51

CNVs: DNA copy number variations. (^∗^ *p* < 0.05) One-way ANOVA, significant difference among means. (^#^ *p* < 0.05) Bonferroni's posttest, in common CNVs versus unique CNVs from the cerebellum.

## Data Availability

The data used to support the findings of this study are available from the corresponding author upon request.

## Supplementary Materials

Supplementary Materials Supplementary Table 1: all DNA copy number variations (CNVs) identified in the frontal cortex and cerebellum. The frequency of deletions and duplications of all unique CNVs (observed exclusively either in the frontal cortex or in the cerebellum) as well as the ones in common to both tissues are presented in their respective sheets. Information about CNV gene content, their length, and genomic position are also presented. Supplementary Table 2: top diseases/function of all networks retrieved from both the frontal cortex and the cerebellum. Ingenuity pathway analysis (IPA) software retrieved the predicted target pathways of all DNA copy number variations (CNVs) identified exclusively in the frontal cortex and cerebellum (unique CNVs).
